# Application of Bidirectional Long Short-Term Memory to Adaptive Streaming for Internet of Autonomous Vehicles

**DOI:** 10.3390/biomimetics8060467

**Published:** 2023-10-01

**Authors:** Chenn-Jung Huang, Kai-Wen Hu, Hao-Wen Cheng

**Affiliations:** 1 Department of Computer Science & Information Engineering, National Dong Hwa University, Shoufeng, Hualien County 974301, Taiwan; 2586611811v@gmail.com; 2 Lookout, Inc., Taipei 110207, Taiwan; drive55555@gmail.com

**Keywords:** bidirectional long short-term memory, biomimicry, intelligence control, autonomous vehicle, streaming service, 6G

## Abstract

It is expected that interconnected networks of autonomous vehicles, especially during peak traffic, will face congestion challenges. Moreover, the existing literature lacks discussions on integrating next-generation wireless communication technologies into connected vehicular networks. Hence, this paper introduces a tailored bandwidth management algorithm for streaming applications of autonomous vehicle passengers. It leverages cutting-edge 6G wireless technology to create a network with high-speed transmission and broad coverage, ensuring smooth streaming application performance. The key features of bandwidth allocation for diverse streaming applications in this work include bandwidth relay and pre-loading of video clips assisted by vehicle-to-vehicle communication. Through simulations, this research effectively showcases the algorithm’s ability to fulfill the bandwidth needs of diverse streaming applications for autonomous vehicle passengers. Specifically, during periods of peak user bandwidth demand, it notably increases the bandwidth accessible for streaming applications. On average, users experience a substantial 55% improvement in the bandwidth they can access. This validation affirms the viability and promise of the proposed approach in efficiently managing the intricate complexities of bandwidth allocation issues for streaming services within the connected autonomous vehicular networks.

## 1. Introduction

In recent years, global car manufacturers have focused on advancing self-driving vehicles. These systems have significantly improved safety and driving convenience. The aim is to achieve fully automated driving in future, where vehicles can independently perceive, evaluate, and respond to various traffic situations, enhancing safety and efficiency. By then, autonomous vehicles will not only be vehicles, but will also become mobile offices and entertainment centers.

In the future, it is conceivable that individuals will find themselves with increased opportunities to engage in various activities while in transit, such as handling emails, engaging in video calls, and enjoying high-quality multimedia streaming. However, efficiently allocating bandwidth for the Internet of autonomous vehicles is a challenge. For instance, videotelephony currently consumes a significant amount of bandwidth and is a crucial part of daily life [1]. Insufficient uplink channel capacity can disrupt videotelephony, which relies on real-time data uploads. Live streaming, another data-intensive application, is increasingly popular. For example, Twitch, a prominent live streaming platform, contributes significantly to United States network traffic [2]. The popularity of streaming services like YouTube and TikTok highlights the appeal of on-demand streaming in everyday life. On-demand streaming has become a primary mode of media consumption and has significantly impacted web traffic patterns [3]. Recently, 360-degree streaming technology has emerged, enabling users to immerse themselves in content from a complete 360-degree perspective. However, it requires a minimum 4K resolution and consumes more bandwidth compared to traditional streaming methods [4].

With the surge in vehicular network traffic, enhancing efficiency becomes paramount. A significant challenge lies in minimizing download delays to enhance the user experience. One viable solution involves the deployment of edge devices for preloading content, a strategy that significantly reduces download times and enhances user satisfaction [5]. Additionally, user devices are experiencing a significant power boost, enabling them to actively engage in edge computing, communication, and storage tasks [6].

Current 5G wireless technology encounters several challenges, including security vulnerabilities, limitations in achieving ultra-low latency, packet loss issues, energy inefficiency, and congestion in high-density areas. These hinder the ability to meet future application demands. Recent literature emphasizes the development of next-generation wireless technologies, as envisioned by experts who aim to achieve advancements in bandwidth, reliability, security, energy efficiency, and ultra-low latency. The overarching objective is to effectively manage a wide range of traffic requirements [7]. Salameh and El Tarhuni [8] introduced groundbreaking technology for the next generation of networks. Their research involved the utilization of previously untapped frequency bands, including those in the visible light and terahertz spectrums, to expand cellular communication standards. An emerging technology, Visible Light Communication (VLC), adheres to eye safety regulations, ensuring secure operation [9]. VLC’s compatibility with LED lighting allows for easy integration, making it a suitable choice for diverse lighting fixtures such as car lights and street lamps [10]. VLC boasts numerous advantages, including a long lifespan, energy efficiency, straightforward installation, user-friendliness, cost-effectiveness facilitated by the use of LEDs [11], and the ability to operate within a license-free spectrum [12]. These characteristics make optical communication a solution that is well suited to vehicle-to-vehicle (V2V) communication.

The terahertz frequency range, which occupies the space between microwave and infrared frequencies, shows promise for achieving low latency, wide bandwidth, and ultra-high-speed data transmission [13]. However, the significant path loss restricts the transmission distance [14]. Moreover, the high cost of terahertz equipment poses a challenge to its widespread deployment [15]. In addressing the limited transmission range inherent to terahertz, the deployment of base stations operating in different frequency bands becomes a necessity. Fortunately, Free Space Optical Communication (FSOC) has emerged as a practical solution. It operates in the near-infrared wavelength spectrum, making it suitable for outdoor use [16]. FSOC offers cost-effective implementation, wide bandwidth, and high-speed data transmission [17]. Therefore, it can be deployed alongside terahertz base stations [18]. However, FSOC’s effectiveness relies on establishing an unobstructed line-of-sight link between transmitters and receivers, limiting its use to point-to-point communication. Nevertheless, non-line-of-sight (NLOS) FSOC [19] addresses this constraint by using diffuse reflectors to disperse light, enabling communication even without a direct line of sight [20].

With the continuous growth of future network traffic, the problem of insufficient bandwidth during peak periods becomes a critical issue. There have been numerous research endeavors dedicated to bandwidth allocation within vehicular networks, aiming to adeptly tackle the intricate challenges linked to the fair distribution of network resources among vehicles. Zhang et al. [21] introduced a Vickrey–Clarke–Groves auction model to tackle resource scheduling challenges for time-sensitive services. They optimized bandwidth allocation for connected autonomous vehicles using a Lagrange relaxation algorithm. Fu et al. [22] introduced a transaction framework based on blockchain technology for communication between vehicles and roadside units (RSUs). They employed a Stackelberg game model to establish secure and optimal bandwidth allocation and pricing strategies between RSUs and vehicles.

Recent literature has witnessed a proliferation of studies presenting bandwidth allocation solutions tailored to multimedia applications for vehicle users. Several of these studies have incorporated caching techniques to mitigate latency. Furthermore, some have delved into the exploration of Space–Air–Ground-Integrated Networks, a concept that integrates satellite networks and unmanned aerial vehicles to enhance communication capabilities. To name a few, Huang et al. [23] proposed a network architecture for Space–Air–Ground Internet of Vehicle Systems, which aimed to ensure quality-of-service-compliant network access for vehicles. They introduced a deployment scheme capable of adapting Access Assistant positions in response to increased network demands stemming from a higher number of vehicles. This scheme also incorporated vehicle information caching to facilitate access decision-making. Xiao et al. [24] introduced a novel 360-degree video caching and delivery framework tailored to advanced edge-enhanced wireless networks. They presented an edge cooperative caching scheme based on multi-agent reinforcement learning and introduced a two-tier base station-multicast group matching mechanism to address collaboration challenges in edge content delivery. Fu [25] delved into video transcoding between different versions, with the base station considering energy consumption when caching video segments. They subsequently proposed a network resource pricing algorithm that factors in the varying usage of caching, base station computing resources, and backhaul links by vehicle users, enhancing network resource utilization flexibility. Ma and Son [26] explored future 5G network-related technologies and internet of vehicle development trends. By incorporating both a 5G base RSU station and a UAV mobile base station, they bolstered network resources for scalable coded video allocation. Additionally, they derived an improved edge caching strategy through an analysis of user mobility. Nevertheless, these studies have yet to fully harness the potential of the latest next-generation wireless communication technologies, explore the possibilities of in-vehicle communication for video transmission, or prioritize real-time applications. As the number of streaming media users continues to grow rapidly, we may encounter bandwidth shortages during peak traffic periods. To address this, we require bandwidth allocation strategies specifically tailored to the unique characteristics of multimedia applications, ensuring that real-time streaming users receive the prioritized bandwidth they require.

Acknowledging the vulnerability of conventional centralized control systems to generate computational and transmission bottlenecks, this study introduces a decentralized computational framework aimed at allocating bandwidth in alignment with user demands.

The primary contributions and novel aspects of this research can be summarized as follows.

- This paper utilizes cutting-edge next-generation wireless technologies, including terahertz micro base stations, NLOS-FSOC small base stations with diffuse reflectors, and existing sub-6GHz macro base stations. Collectively, these technologies address passenger bandwidth requirements, establishing a network characterized by low latency, extensive coverage, high-speed connectivity, and exceptional reliability.
- In our research, we have considered the distinct characteristics of various applications. Specifically, when passengers use real-time applications in situations with limited bandwidth, our algorithm prioritizes allocating the originally designated bandwidth for non-real-time applications to passengers engaged in activities like videotelephony and live streaming. Additionally, we have implemented V2V communication through VLC to enable bandwidth sharing among vehicles within the fleet, allowing for the relay of bandwidth from base stations in other segments. In cases where the required bandwidth remains insufficient, we employ dynamic streaming resolution adjustments, enabling playback with lower bandwidth requirements.
- In scenarios where there is insufficient bandwidth for non-real-time streaming, this research leverages less congested base stations and autonomous vehicles to assist with the preloading of video segments. When autonomous vehicles converge on the same road segment, VLC is employed for V2V communication to transmit preloaded streaming segments. To determine the arrival times of autonomous vehicles on each road segment, the study utilizes bidirectional long short-term memory (BILSTM) [27] to estimate average vehicle speeds. Consequently, this proposed approach maximizes the benefits of V2V communication by efficiently utilizing less congested base station bandwidth and reducing the strain on congested segment base stations. It is worth noting that the loading of the backbone network is not significantly affected because the emerging solution for ultra-gigabit optical access backbone [28] offers considerably higher bandwidth capacity compared to the current backbone infrastructure.

## 2. Adaptive Streaming Algorithm for Internet of Autonomous Vehicles

**Figure 1:**
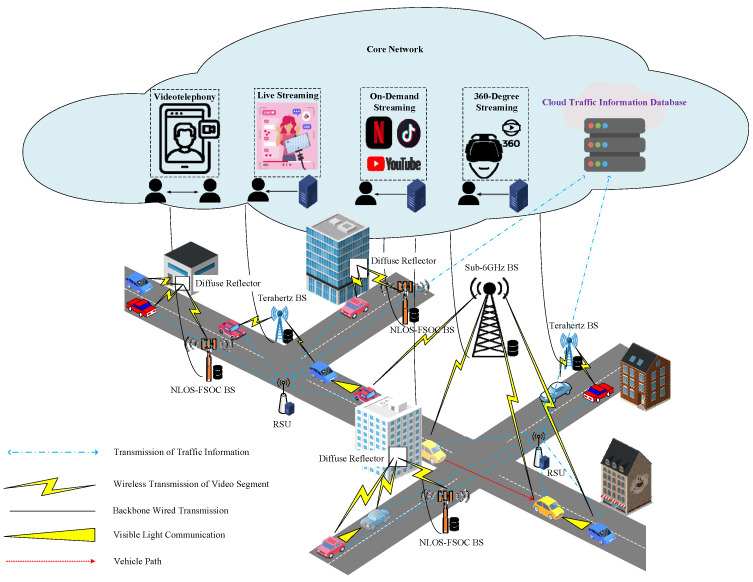
Illustration of streaming services in the Internet of autonomous vehicles.

As illustrated in Figure 1, this study introduces a decentralized computational framework aimed at allocating bandwidth in alignment with the requirements of autonomous vehicle passengers. The management of bandwidth allocation for distinct road segments is carried out by the RSUs strategically positioned along these specific segments. Autonomous vehicle passengers plan their routes in advance, and as autonomous vehicles traverse their designated paths, the RSU associated with each segment orchestrates bandwidth allocation in alignment with the streaming service application requisites of the corresponding autonomous vehicle passengers. In scenarios where the streaming service dedicated to autonomous vehicle passengers falls short of fulfilling their bandwidth demands while navigating through congested road segments, the autonomous vehicle has the capability to solicit assistance from the overseeing RSU responsible for that specific segment. This support encompasses procuring the necessary streaming service bandwidth. The basis of this study revolves around categorizing the multimedia streaming applications employed by passengers in autonomous vehicles into four distinct types: bidirectional videotelephony, unidirectional live streaming, on-demand streaming, and 360-degree streaming.

**Figure 2:**
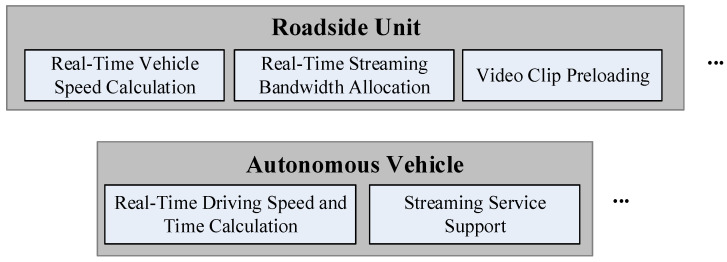
Schematic diagram of adaptive streaming for the Internet of autonomous vehicles.

Figure 2 illustrates the system architecture of the proposed algorithm, which is meticulously designed to meet the bandwidth requirements of multimedia streaming applications used by passengers in autonomous vehicles. Before embarking on the journey, an autonomous vehicle activates the “Real-Time Driving Speed and Time Calculation” module integrated within the On-Board Unit (OBU) to plan the driving route. This route information is then transmitted to the RSUs responsible for supervising the individual segments along the designated route.

Each RSU hosts the “Real-Time Vehicle Speed Calculation” module, which estimates the average vehicle speed as the autonomous vehicle progresses through the managed segments within specific time intervals. It is worth noting that in recent academic research, the Bidirectional Long Short-Term Memory (BILSTM) architecture has garnered significant attention for its effectiveness in predicting vehicle traffic flow across various road segments, as highlighted in previous studies [29,30,31,32]. Given the substantial impact of traffic flow patterns on the driving speed of autonomous vehicles, this module utilizes the BILSTM model introduced in [27] to compute the average vehicle speed.

In cases where streaming service applications are needed during the vehicle’s journey, the OBU’s “Streaming Service Support” module establishes a connection with the streaming service provider. This connection enables the acquisition of the relevant requirements and specifications associated with the application. Guided by the service quality criteria predefined by the autonomous vehicle passenger and assisted by the most up-to-date traffic patterns and base station bandwidth data provided by the RSUs for each road segment, the module dynamically adjusts the bandwidth requirements and quality tailored to the streaming service.

Following this, the OBU will communicate the obtained bandwidth requirements for streaming service applications to the RSUs as the autonomous vehicle progresses through various road segments during the usage of these applications. Subsequently, each RSU will allocate the necessary bandwidth for the streaming service application based on its specific demands using the “Real-Time Streaming Bandwidth Allocation” and “Video Clip Preloading” modules. These modules leverage the three categories of base stations mentioned earlier: terahertz micro base stations, NLOS-FSOC small base stations with diffusers, and sub-6GHz macro base stations. These base stations are employed to fulfill the essential bandwidth requirements for the streaming service applications as the autonomous vehicle traverses its managed route segments. In this framework, it is assumed that each RSU maintains a server to record the supply and demand information for streaming applications across distinct time intervals within its managed segments. Consequently, the base stations situated along the route segments will provide the required bandwidth, aligning with the anticipated streaming service quality standards and specifications preset by autonomous vehicle passengers.

If the coverage area of a managed route segment’s base station falls short of delivering the required bandwidth, the RSU will step in to ensure that the streaming service quality standards are met, tailoring its approach to the characteristics of the streaming service application. For videotelephony and live streaming, bandwidth allocation can be facilitated through V2V communication with other nearby roadside base stations. Conversely, for on-demand streaming and 360-degree streaming, a video clip preloading mechanism is employed. 

In scenarios of insufficient bandwidth for on-demand streaming or 360-degree streaming, the RSU examines other autonomous vehicles arriving at the same road segment and time as the streaming-enabled autonomous vehicles. It analyzes their travel routes to identify whether there are base stations capable of providing the required bandwidth for preloading video clips. Upon finding a suitable base station, the managing RSU of that segment coordinates with the supported autonomous vehicles to preload video clips from the base station’s coverage area when they traverse the corresponding road segment. These preloaded video clips are stored in the storage facilities of the designated autonomous vehicles. Consequently, when both the autonomous vehicle equipped with a specific preloaded video clip and the one engaged in streaming reach the same road segment, the V2V communication is employed to transmit the preloaded video clip to the autonomous vehicle passenger who is streaming the content. Furthermore, it is important to note that one of the primary objectives of this research is to evaluate the effectiveness of the pre-loading strategy via V2V communication proposed in this work. Therefore, it is assumed that each required video clip is cached at the base station for access before the conveying autonomous vehicles arrive in the vicinity of the base station to initiate the pre-loading process. 

Passengers in autonomous vehicles who utilize streaming services also conduct intermittent evaluations of the streaming service quality while it is being used. In situations where modifications to the streaming service quality are deemed necessary, such as reducing it during periods of high demand, the vehicle will establish communication with the “Real-Time Streaming Bandwidth Allocation” or “Video Clip Preloading” module located at the managed Roadside Unit (RSU). This interaction will prompt the respective module to undertake the subsequent task of adjusting the streaming bandwidth as required.

The detailed description of each module shown in Figure 2 is as follows.

### 2.1. Real-Time Vehicle Speed Calculation at a RSU

To predict autonomous vehicle driving speeds, the BILSTM model mentioned earlier is utilized with historical data. This architecture processes information simultaneously in both the forward and backward directions, with the overarching goal of enhancing the accuracy of driving speed predictions for autonomous vehicles.

**Figure 3:**
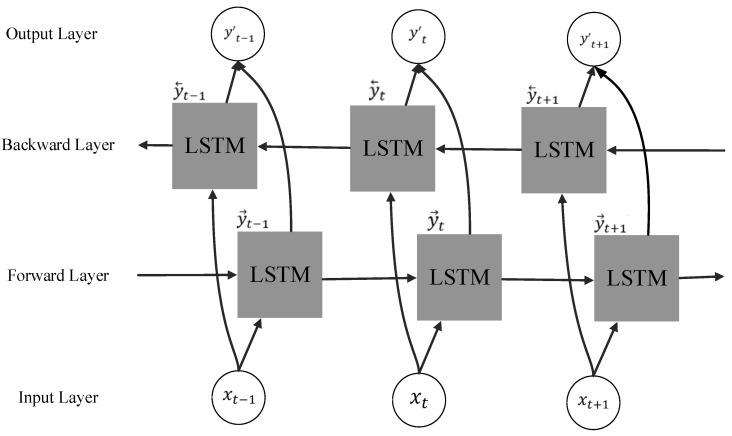
A BILSTM architecture.

The BILSTM model, as introduced in [27] and depicted in Figure 3, serves the purpose of estimating the average speed of autonomous vehicles as they enter specific road segments. The BILSTM framework builds upon the foundation of the Long Short-Term Memory (LSTM) model [33] and incorporates two distinct LSTM components. One component specializes in processing forward time sequences, while the other is tailored for handling backward time sequences. This dual-component configuration allows for the simultaneous integration of bidirectional information, effectively addressing the limitations encountered in conventional LSTM models due to data scarcity. The outcome is a noticeable improvement in prediction accuracy, as demonstrated by findings in the literature [34].

The conventional LSTM architecture comprises an input layer, a recurrent hidden layer composed of memory blocks as foundational components, and an output layer. Within each memory block, interconnected memory cells preserve temporal states. Moreover, three adaptable multiplication gate cells—the input gate, output gate, and forget gate—oversee the information flow within the block. In the context of the unidirectional LSTM model, it predicts information for a specific future time by utilizing historical data in the following manner:

(1) $$f_{t}=\sigma \left(W_{f}\cdot \left[y_{t-1},x_{t}\right]+b_{f}\right)$$

(2) $$i_{t}=\sigma \left(W_{i}\cdot \left[y_{t-1},x_{t}\right]+b_{i}\right)$$

(3) $$s_{t}=\tanh\left(W_{s}\left[y_{t-1},x_{t}\right]+b_{s}\right)$$

(4) $$C_{t}=f_{t}C_{t-1}+i_{t}s_{t}$$

(5) $$o_{t}=\sigma \left(W_{o}\cdot \left[y_{t-1},x_{t}\right]+b_{o}\right)$$

(6) $$y_{t}=o_{t}\tanh\left(C_{t}\right)$$

where $f_{t}$ and $i_{t}$ denote the forget and input gates of the LSTM, respectively. $y_{t-1}$ is the previous output result. $x_{t}$ stands for the input at time *t*, and $\sigma \left(\cdot \right)$ represents the sigmoid activation function. $W_{f}$ and $W_{i}$ denote the weights of the forget and input gates, respectively, and $b_{f}$ and $b_{i}$ are the bias vectors of the forget and input gates, respectively. The input values $x_{t}$ and $y_{t-1}$ play a role in deciding the relevant updates to the cell state, utilizing the sigmoid activation function of the input gate. This process leads to the generation of a new cell candidate state, denoted as $s_{t}$, which is achieved through the $tanh\left(\cdot \right)$ activation function of the input gate.$W_{s}$ and $b_{s}$ denote the weights and bias of the cell candidate state, respectively. By utilizing the forgetting gate and the input gate, the cell state $C_{t-1}$ undergoes an update to become $C_{t}$. Following this update, the output gate $o_{t}$ is activated using the sigmoid function. Here, $f_{t}C_{t-1}$ represents the information to be forgotten, and $i_{t}s_{t}$ stands for the information to be added. In the final step, the new cell state $C_{t}$, after undergoing the tanh(∙) transformation, is multiplied by the output gate $o_{t}$, resulting in the output value $y_{t}$.

(7) $${\vec{y}}_{t}=LSTM\left.\left(x_{t},\;{\vec{y}}_{t-1}\right.\right)$$

(8) $${\overset{\leftarrow }{y}}_{t}=LSTM\left.\left(x_{t},\;{\overset{\leftarrow }{y}}_{t-1}\right.\right)$$

(9) $${y\prime }_{t+1}=OL\left.\left(W_{\vec{y\prime }}{\vec{y}}_{t}+W_{\overset{\leftarrow }{y\prime }}{\overset{\leftarrow }{y}}_{t}+b_{y\prime }\right.\right)$$

Here, ${\vec{y}}_{t}$ and ${\overset{\leftarrow }{y}}_{t}$ represent the output results of the forward and backward LSTM, respectively. The $LSTM\left.\left(\cdot \right.\right)$ mentioned in Equations (8) and (9) denotes the unidirectional LSTM. ${\vec{y}}_{t-1}$ denotes the output state from the prior instance of forward propagation along the timeline, while ${\overset{\leftarrow }{y}}_{t-1}$ represents the output state from the preceding moment of backward propagation along the timeline. OL$\left.\left(\cdot \right.\right)$ denotes the activation function of the output layer. $W_{\vec{y\prime }}$ and $W_{\overset{\leftarrow }{y\prime }}$ stand for the weight matrices of the calculated output result ${y\prime }_{t}$, and $b_{y\prime }$ is the output bias. 

As mentioned above, a BILSTM is employed, consisting of a dual-layered LSTM structure that simultaneously transmits information in both the forward and backward directions. Finally, the two output state variables are concatenated to form the ultimate output. To be specific, the BILSTM takes input sequences $x_{t-1}$, $x_{t}$, and $x_{t+1}$, and feeds them into the forward and backward LSTMs. In this study, the average velocity time series at time slots (t−1), *t*, and (t+1) are utilized as inputs to acquire the outputs from the LSTM in different directions. These outputs are then merged to obtain the final prediction result. 

The overall time complexity of training a BILSTM network over a fixed number of epochs is typically O(E·L·N·M), where *E* denotes the number of training epochs, *L* stands for the number of layers, *N* represents the sequence length, and *M* is the number of cells per layer [27]. 

### 2.2. Real-Time Driving Speed and Time Calculation for an Autonomous Vehicle

This module is initiated by autonomous vehicle passengers prior to the vehicle’s departure. The passengers input the departure location, time, and intended destination, initiating the vehicle’s operation. Subsequently, the vehicle retrieves traffic data and utilizes the Dijkstra algorithm [35] to calculate the most efficient driving path from the point of origin to the final destination. The estimation is based on average travel times for individual road segments. 

Autonomous vehicle passengers have the flexibility to spontaneously modify their travel plans. Furthermore, the arrival times and speeds of autonomous vehicles on various road segments can be influenced by real-time traffic conditions, which can vary over time and deviate from the initially estimated times and speeds. This module periodically receives updates on traffic conditions for different road segments and gathers relevant data regarding the vehicle’s average speed from RSUs. It then proceeds to reevaluate the expected arrival times of autonomous vehicles at each road segment to align with the updated conditions. If the disparity between the revised arrival times and the originally projected times is significant, this module transmits the revised arrival information to the RSUs tasked with monitoring the respective road sections.

The steps executed by this module are as follows:

Step 1: Before the autonomous vehicle sets off, it retrieves road traffic data. Once the departure location, time, and destination are inputted, this module utilizes the Dijkstra algorithm [35] to compute the most efficient path from the initial location to the endpoint. This computation considers the average travel duration for each segment of the roadway as the foundation for travel cost.
Step 2: Estimate the anticipated segment-specific arrival times using real-time traffic data from the autonomous vehicle’s database.
  (10) $${at}_{p_{i+1}^{\sigma }}={at}_{p_{i}^{\sigma }}+\frac{{sl}_{p_{i}^{\sigma },p_{i+1}^{\sigma }}}{{sp}_{p_{i}^{\sigma },p_{i+1}^{\sigma }}\left({at}_{p_{i}^{\sigma }}\right)},1\leq i<h^{\sigma },$$
  where $p_{1}^{\sigma }$, $p_{i}^{\sigma }$, and $p_{h^{\sigma }}^{\sigma }$, respectively, represent the starting point of autonomous vehicle σ, the point where the road segment linking $p_{i}^{\sigma }$ to $p_{i+1}^{\sigma }$, and the final destination of σ’s route. ${at}_{p_{i}^{\sigma }}$ is the time σ arrives at $p_{i}^{\sigma }$. ${sl}_{p_{i}^{\sigma },p_{i+1}^{\sigma }}$ denotes the length of the segment connecting $p_{i}^{\sigma }$ and $p_{i+1}^{\sigma }$. ${sp}_{p_{i}^{\sigma },p_{i+1}^{\sigma }}\left({at}_{p_{i}^{\sigma }}\right)$ stands for the average vehicle speed through the segment connecting $p_{i}^{\sigma }$ and $p_{i+1}^{\sigma }$ after the autonomous vehicle arrives at $p_{i}^{\sigma }$ at time ${at}_{p_{i}^{\sigma }}$.
Step 3: The calculated arrival times for each road segment, along with the travel route of the autonomous vehicle, are sent to the RSUs responsible for managing those segments.
Step 4: This component operates in the background. In the event of a modification to the itinerary by the autonomous vehicle passenger, the process reverts to Step 1 to recompute the travel route.
Step 5: At predetermined intervals established by the system, this module retrieves the most recent road traffic data from the overseeing RSUs situated along the route. Subsequently, it proceeds to recalculate the estimated arrival times for each segment of the travel route.
  (11) $${at\prime }_{p_{j+1}^{\sigma }}={at\prime }_{p_{j}^{\sigma }}+\frac{{sl}_{p_{j}^{\sigma },p_{j+1}^{\sigma }}}{{sp\prime }_{p_{j}^{\sigma },p_{j+1}^{\sigma }}\left({at\prime }_{p_{j}^{\sigma }}\right)},\kappa^{\sigma }\leq j<h^{\sigma }$$
  (12) $${at\prime }_{p_{\kappa^{\sigma }}^{\sigma }}={ct}^{\sigma }+\frac{{rsl}_{p_{\kappa^{\sigma }-1}^{\sigma },p_{\kappa^{\sigma }}^{\sigma }}}{{sp\prime }_{p_{\kappa^{\sigma }-1}^{\sigma },p_{\kappa^{\sigma }}^{\sigma }}\left({at\prime }_{p_{\kappa^{\sigma }-1}^{\sigma }}\right)}$$
  (13) $$p_{1}^{\sigma }<p_{\kappa^{\sigma }}^{\sigma },$$
  where ${ct}^{\sigma }$ represents the current time, ${at\prime }_{p_{j}^{\sigma }}$ denotes the recalculated arrival time of σ at $p_{j}^{\sigma }$, and ${\rho \prime }_{p_{j}^{\sigma },p_{j+1}^{\sigma }}\left({at\prime }_{p_{j}^{\sigma }}\right)$ stands for the traffic flow of the segment connecting $p_{j}^{\sigma }$ and $p_{j+1}^{\sigma }$ at time ${at\prime }_{p_{j}^{\sigma }}$. Significantly, $\kappa^{\sigma }$ designates the initiation point of the subsequent road segment that σ will be approaching. ${sp\prime }_{p_{\kappa^{\sigma }-1}^{\sigma },p_{\kappa^{\sigma }}^{\sigma }}\left({at\prime }_{p_{\kappa^{\sigma }-1}^{\sigma }}\right)$ denotes the driving speed of σ on the current segment, and ${sp\prime }_{p_{j}^{\sigma },p_{j+1}^{\sigma }}\left({at\prime }_{p_{j}^{\sigma }}\right)$ represents the average vehicle speed of σ passing through the segment connecting $p_{i}^{\sigma }$ and $p_{i+1}^{\sigma }$ after reaching ${at\prime }_{p_{j}^{\sigma }}$. In this context, ${rsl}_{p_{\kappa^{\sigma }-1}^{\sigma },p_{\kappa^{\sigma }}^{\sigma }}$ represents the remaining length of the current route segment that σ is currently traversing.
Step 6: Compute whether the arrival time of the autonomous vehicle at each segment surpasses each time slot interval set by the system:
  (14) $${dat}_{p_{j}^{\sigma }}^{\sigma }=\left\{\begin{array}{cc} 1 & if\;\left|{at\prime }_{p_{j}^{\sigma }}-{at}_{p_{j}^{\sigma }}\right|\geq ∆\\ 0 & else \end{array}\right.,\;\kappa^{\sigma }\leq j<h^{\sigma }$$
  where ∆ represents each time slot interval.
Step 7: If the previously computed ${dat}_{p_{j}^{\sigma }}^{\sigma }$ is equal to 1, the relevant RSU overseeing the segment is notified about the updated arrival time for the segment traversal.
Step 8: Before the autonomous vehicle reaches its intended destination, we return to Step 4 and proceed with the execution.

The primary computational overhead in this module occurs during Step 1, which executes the Dijkstra algorithm [35]. The computational complexity of the Dijkstra algorithm, as explained in [35], is O(|*E*| log |*V*|), where |*E*| and |*V*| represent the total number of road segments and intersections in this module, respectively.

### 2.3. Streaming Service Support for an Autonomous Vehicle

When a passenger in an autonomous vehicle initiates a streaming service, this module initiates a connection with the server of the streaming service provider. The purpose of this connection is to collect the necessary requirements and specifications for the streaming service. Subsequently, the module engages with the RSUs positioned along the vehicle’s route. The primary objective is to assess whether the bandwidth supplied by the base stations, which cover the segments through which the vehicle travels, aligns with the streaming application’s demands. During this evaluation, the module carefully considers the passenger’s predefined quality preferences for their streaming experience. The aim is to ensure that the selected base stations can adequately support the desired streaming quality for the duration of travel along each road segment.

In cases of bandwidth insufficiency during peak hours, this module will transmit bandwidth requirement-related information of the autonomous vehicle passenger’s streaming service application to the RSUs positioned along the various road segments traversed during the application’s usage. These RSUs will then, based on the streaming service application’s characteristics and specification demands, undertake bandwidth allocation or preload planning of video segments for the streaming service application as the autonomous vehicle journeys through the managed route segments. Additionally, this module will also respond to bandwidth supply–demand imbalances that arise during peak periods. It will proactively adjust the bandwidth requirements and quality of the streaming service as necessary.

The steps executed by this module are as follows:

Step 1: Upon activation of the streaming service application by the autonomous vehicle passenger, the module interacts with the streaming service provider’s server to acquire the relevant requirements and specifications of the application. Concurrently, this module establishes communication with RSUs positioned along the predetermined route of the autonomous vehicle. The aim is to retrieve information regarding the minimum bandwidth that the base stations can offer as the vehicle traverses through individual road segments.
Step 2: In the event that the streaming service application pertains to an on-demand streaming service, the process advances to Step 9 for continued execution.
Step 3: Using the duration of the autonomous vehicle’s presence on each road segment and considering the streaming resolution chosen by the passenger, this module computes the dynamic bandwidth needed for each time interval throughout the vehicle’s journey. It subsequently applies the following equations to determine whether the bandwidth from the surrounding base stations meets the necessary criteria for uninterrupted real-time streaming.
  (15) $${dub}_{t_{j}^{\sigma ,\alpha }}^{\sigma ,\alpha }=\gamma^{\sigma ,\alpha }\cdot \left[\sum_{\vartheta }{ub}_{t_{j}^{\sigma ,\alpha }}^{\vartheta ,\sigma ,\alpha }-\underline{UR}\left({uvr}_{t_{j}^{\sigma ,\alpha }}^{\sigma ,\alpha }\right)\right],\;1\leq j\leq e^{\sigma ,\alpha }$$
  (16) $$u{rsu}_{t_{j}^{\sigma ,\alpha }}^{\sigma ,\alpha }=\left\{\begin{array}{cc} {rsu}_{p_{i}^{\sigma }} & if\;{dub}_{t_{j}^{\sigma ,\alpha }}^{\sigma ,\alpha }<0,\;{at}_{p_{i}^{\sigma }}\leq t_{j}^{\sigma ,\alpha }\leq {at}_{p_{i+1}^{\sigma }},\;1\leq j\leq e^{\sigma ,\alpha },1\leq i<h^{\sigma }\\ 0 & else \end{array}\;\right.$$
  (17) $${ddb}_{t_{j}^{\sigma ,\alpha }}^{\sigma ,\alpha }=\sum_{\vartheta }{db}_{t_{j}^{\sigma ,\alpha }}^{\vartheta ,\sigma ,\alpha }-\underline{DR}\left({dvr}_{t_{j}^{\sigma ,\alpha }}^{\sigma ,\alpha }\right),\;\;1\leq j\leq e^{\sigma ,\alpha }\;$$
  (18) $$d{rsu}_{t_{j}^{\sigma ,\alpha }}^{\sigma ,\alpha }=\left\{\begin{array}{cc} {rsu}_{p_{i}^{\sigma }} & if\;{ddb}_{t_{j}^{\sigma ,\alpha }}^{\sigma ,\alpha }<0,\;{at}_{p_{i}^{\sigma }}\leq t_{j}^{\sigma ,\alpha }\leq {at}_{p_{i+1}^{\sigma }},\;1\leq j\leq e^{\sigma ,\alpha },1\leq i<h^{\sigma }\\ 0 & else \end{array}\;\right.$$
  (19) $${uvr}_{t_{j}^{\sigma ,\alpha }}^{\sigma ,\alpha }={dvr}_{t_{j}^{\sigma ,\alpha }}^{\sigma ,\alpha }={vr}_{q^{\sigma ,\alpha }}^{\sigma ,\alpha },\;1\leq j<e^{\sigma ,\alpha },\;1\leq q^{\sigma ,\alpha }\leq {\bar{q}}^{\alpha }$$
  (20) $$t_{j+1}^{\sigma ,\alpha }=t_{j}^{\sigma ,\alpha }+∆,\;1\leq j<e^{\sigma ,\alpha }$$
  (21) $${at}_{p_{1}^{\sigma }}\leq t_{1}^{\sigma ,\alpha }\leq t_{j}^{\sigma ,\alpha }\leq t_{e^{\sigma ,\alpha }}^{\sigma ,\alpha }\leq {at}_{p_{i+1}^{\sigma }},\;1\leq j\leq e^{\sigma ,\alpha },$$
  where the binary flag $\gamma^{\sigma ,\alpha }$ indicates whether the real-time streaming occurs via videotelephony. ${ub}_{t_{j}^{\sigma ,\alpha }}^{\vartheta ,\sigma ,\alpha }$ and ${db}_{t_{j}^{\sigma ,\alpha }}^{\vartheta ,\sigma ,\alpha }$, respectively, denote the upload and download bandwidth that base station ϑ can allocate to real-time streaming application α when σ passes through its coverage during time slot $t_{j}^{\sigma ,\alpha }$. ${vr}_{q^{\sigma ,\alpha }}^{\sigma ,\alpha }$ denotes the streaming service resolution requirement set by the autonomous vehicle passenger at level $q^{\sigma ,\alpha }$, with streaming service resolution divided into levels ${\bar{q}}^{\alpha }$. ${uvr}_{t_{j}^{\sigma ,\alpha }}^{\sigma ,\alpha }$ and ${dvr}_{t_{j}^{\sigma ,\alpha }}^{\sigma ,\alpha }$, respectively, representing the upload and download streaming resolutions for α, while $\underline{UR}\left({uvr}_{t_{j}^{\sigma ,\alpha }}^{\sigma ,\alpha }\right)$ and $\underline{DR}\left({dvr}_{t_{j}^{\sigma ,\alpha }}^{\sigma ,\alpha }\right)$ indicate the upload and download bandwidth requirements for the uploading resolution ${uvr}_{t_{j}^{\sigma ,\alpha }}^{\sigma ,\alpha }$ and downloading resolution ${dvr}_{t_{j}^{\sigma ,\alpha }}^{\sigma ,\alpha }$ of α, respectively. $t_{1}^{\sigma ,\alpha }$ and $t_{e^{\sigma ,\alpha }}^{\sigma ,\alpha }$, respectively, denote the start and end times for α. ${rsu}_{t_{j}^{\sigma ,\alpha }}^{\sigma ,\alpha }$ signifies the RSU managing road segment $p_{i}^{\sigma }$ that σ passes through during time slot $t_{j}^{\sigma ,\alpha }$. If the values of ${dub}_{t_{j}^{\sigma ,\alpha }}^{\sigma ,\alpha }$ or ${ddb}_{t_{j}^{\sigma ,\alpha }}^{\sigma ,\alpha }$ fall below zero, it indicates that the upload or download bandwidth of the base station for that road segment cannot meet α’s minimum bandwidth requirements. In such instances, $u{rsu}_{t_{j}^{\sigma ,\alpha }}^{\sigma ,\alpha }$ and $d{rsu}_{t_{j}^{\sigma ,\alpha }}^{\sigma ,\alpha }$ are used to represent the RSU overseeing the road segment $p_{i}^{\sigma }$, where the autonomous vehicle passenger encounters this insufficiency in upload or download bandwidth.
Step 4: If all the values of $u{rsu}_{t_{j}^{\sigma ,\alpha }}^{\sigma ,\alpha }$ and $d{rsu}_{t_{j}^{\sigma ,\alpha }}^{\sigma ,\alpha }$ calculated in the previous step are zero, it suggests that the base stations along the autonomous vehicle’s route can all fulfill the user’s minimum bandwidth needs for real-time streaming. In this case, the module notifies the respective RSUs of the required bandwidth for the autonomous vehicle passing through the managed road segment. Otherwise, we proceed to Step 6 to continue the execution.
Step 5: This component functions in the background mode of execution. Should the itinerary of the autonomous vehicle passenger be altered or if notable deviations occur between the anticipated arrival times at various road segments and the originally projected times, this module reverts to Step 3 to continue execution.
Step 6: In cases where the values of $u{rsu}_{t_{j}^{\sigma ,\alpha }}^{\sigma ,\alpha }$ or $d{rsu}_{t_{j}^{\sigma ,\alpha }}^{\sigma ,\alpha }$ are non-zero, this indicates that the upload or download bandwidth from the base stations within the road segment at the time slot $t_{j}^{\sigma ,\alpha }$ is insufficient to satisfy the real-time streaming service’s minimum bandwidth requirement. In such situations, this module notifies the relevant managing RSU, represented as $u{rsu}_{t_{j}^{\sigma ,\alpha }}^{\sigma ,\alpha }$ or $d{rsu}_{t_{j}^{\sigma ,\alpha }}^{\sigma ,\alpha }$. Upon receiving this notification, the managing RSU triggers the “Real-Time Streaming Bandwidth Allocation” process to facilitate the allocation of the deficient bandwidth required for the real-time streaming service of the autonomous vehicle passenger during the time slot $t_{j}^{\sigma ,\alpha }$. Following this adjustment, the RSU conveys the outcomes of the bandwidth allocation back to this module for further processing.
Step 7: If the allocation of bandwidth in the preceding step meets the minimum bandwidth requirement for the real-time streaming service, we return to Step 5 and continue the execution. If not, continue to the next step.
Step 8: When the base stations along a specific road segment cannot fulfill the real-time streaming service’s minimum bandwidth requirements, and if the streaming service resolution for that segment has already been lowered to the predetermined lower limit, this module informs the autonomous vehicle passenger of the situation. Then, the process returns to Step 5 to continue with further execution.
  On the other hand, if the streaming service resolution for that segment has not yet reached the predefined lower limit, the module advances to diminish the quality prerequisites for the real-time streaming service of the autonomous vehicle passenger, in accordance with the following equations:
  (22) $${uvr}_{t_{j}^{\sigma ,\alpha }}^{\sigma ,\alpha }=\left\{\begin{array}{cc} {vr}_{q^{\sigma ,\alpha }-1}^{\sigma ,\alpha } & if\;u{rsu}_{t_{j}^{\sigma ,\alpha }}^{\sigma ,\alpha }>0,\;q^{\sigma ,\alpha }>1,\;{at}_{p_{i}^{\sigma }}\leq t_{j}^{\sigma ,\alpha }\leq {at}_{p_{i+1}^{\sigma }},\;1\leq j\leq e^{\sigma ,\alpha },1\leq i<h^{\sigma }\\ {\underline{vr}}^{\alpha } & else \end{array}\right.$$
  (23) $${dvr}_{t_{j}^{\sigma ,\alpha }}^{\sigma ,\alpha }=\left\{\begin{array}{cc} {vr}_{q^{\sigma ,\alpha }-1}^{\sigma ,\alpha } & if\;d{rsu}_{t_{j}^{\sigma ,\alpha }}^{\sigma ,\alpha }>0,\;q^{\sigma ,\alpha }>1,\;{at}_{p_{i}^{\sigma }}\leq t_{j}^{\sigma ,\alpha }\leq {at}_{p_{i+1}^{\sigma }},\;1\leq j\leq e^{\sigma ,\alpha },1\leq i<h^{\sigma }\\ {\underline{vr}}^{\alpha } & else \end{array}\right.,$$
  where ${\underline{vr}}^{\alpha }$ represents the system-defined lower limit for the streaming resolution of α. This module notifies the autonomous vehicle passenger and returns to Step 3 for further execution.
Step 9: If the autonomous vehicle passenger’s streaming application involves on-demand streaming or 360-degree streaming, this module calculates whether the autonomous vehicle can smoothly play each video clip of the streaming service during its journey. This means that the video clips can be preloaded to the onboard storage facilities of the vehicle before their playback. This module evaluates whether the base stations along the route can meet the requirements for preloading each video clip of the streaming service using the following equations:
  (24) $$\sum_{\mu }\left(∆\cdot \sum_{\vartheta }{db}_{\tau_{s^{\sigma ,\alpha }}^{\sigma ,\alpha }+\mu \cdot ∆}^{\vartheta ,\sigma ,\alpha }\right){\geq SS}_{s^{\sigma ,\alpha }}^{\sigma ,\alpha }\left({sbr}_{s^{\sigma ,\alpha }}^{\sigma ,\alpha },{sel}_{s^{\sigma ,\alpha }}^{\sigma ,\alpha }\right),\;\;1\leq s^{\sigma ,\alpha }\leq S^{\sigma ,\alpha }$$
  (25) $${pt}_{1}^{\sigma ,\alpha }\leq \tau_{s^{\sigma ,\alpha }}^{\sigma ,\alpha }<\tau_{s^{\sigma ,\alpha }}^{\sigma ,\alpha }+\mu \cdot ∆<{pt}_{s^{\sigma ,\alpha }}^{\sigma ,\alpha },\;1\leq s^{\sigma ,\alpha }\leq S^{\sigma ,\alpha }$$
  (26) $${sbr}_{s^{\sigma ,\alpha }}^{\sigma ,\alpha }={br}_{q^{\sigma ,\alpha }}^{\sigma ,\alpha },\;1\leq s^{\sigma ,\alpha }\leq S^{\sigma ,\alpha }$$
  (27) $${\;pt}_{s^{\sigma ,\alpha }-1}^{\sigma ,\alpha }\leq {pt}_{s^{\sigma ,\alpha }}^{\sigma ,\alpha },\;\;1<s^{\sigma ,\alpha }\leq S^{\sigma ,\alpha }$$
  (28) $${at}_{p_{1}^{\sigma }}\leq {pt}_{1}^{\sigma ,\alpha }$$
  (29) $${buf}_{\tau_{s^{\sigma ,\alpha }}^{\sigma ,\alpha }}^{\sigma }+{SS}_{s^{\sigma ,\alpha }}^{\sigma ,\alpha }\left({sbr}_{s^{\sigma ,\alpha }}^{\sigma ,\alpha },{sel}_{s^{\sigma ,\alpha }}^{\sigma ,\alpha }\right)\leq {\bar{buf}}^{\sigma },\;\;{pt}_{1}^{\sigma ,\alpha }\leq \tau_{s^{\sigma ,\alpha }}^{\sigma ,\alpha }<{pt}_{s}^{\sigma ,\alpha },\;\;\;\;1<s^{\sigma ,\alpha }\leq S^{\sigma ,\alpha },$$
  In this context, $S^{\sigma ,\alpha }$ represents the number of video clips in on-demand streaming or 360-degree streaming. $\tau_{s^{\sigma ,\alpha }}^{\sigma ,\alpha }$ signifies the time at which video clip $s^{\sigma ,\alpha }$ is preloaded. ${pt}_{s^{\sigma ,\alpha }}^{\sigma ,\alpha }$ denotes the playback time of video clip *s*. ${SS}_{s^{\sigma ,\alpha }}^{\sigma ,\alpha }\left(\cdot \right)$ represents the size of video clip $s^{\sigma ,\alpha }$, while ${sbr}_{s^{\sigma ,\alpha }}^{\sigma ,\alpha }$ and ${sel}_{s^{\sigma ,\alpha }}^{\sigma ,\alpha }$ correspond to the resolution and duration of video clip *s*, respectively. ${db}_{\tau_{s^{\sigma ,\alpha }}^{\sigma ,\alpha }}^{\vartheta ,\sigma ,\alpha }$ indicates the bandwidth allocated by base station ϑ to video clip $s^{\sigma ,\alpha }$ at time $\tau_{s^{\sigma ,\alpha }}^{\sigma ,\alpha }$ during a time interval ∆. μ represents the number of consecutive time slots used to download video clips commencing at time $\tau_{s^{\sigma ,\alpha }}^{\sigma ,\alpha }$. ${pt}_{1}^{\sigma ,\alpha }$ and ${pt}_{s^{\sigma ,\alpha }}^{\sigma ,\alpha }$, respectively, stand for the starting and ending times for on-demand streaming or 360-degree streaming. ${br}_{q^{\sigma ,\alpha }}^{\sigma ,\alpha }$ signifies the video screen resolution requirement set by the autonomous vehicle passenger for level $q^{\sigma ,\alpha }$. ${buf}_{\tau_{s^{\sigma ,\alpha }}^{\sigma ,\alpha }}^{\alpha }$ represents the remaining buffer capacity of σ at time $\tau_{s^{\sigma ,\alpha }}^{\sigma ,\alpha }$. ${\bar{buf}}^{\sigma }$ indicates the size of σ’s buffer.
Step 10: In scenarios where the calculated bandwidth for any video clip, whether intended for on-demand streaming or 360-degree streaming, falls below the minimum required download bandwidth threshold as the video clip traverses a road segment, this module activates an alert targeted at the supervising RSU of the corresponding road segment. The objective of this alert is to foster synchronization among other autonomous vehicles, prompting them to preload the essential video clips essential to the streaming service. Upon receipt of this alert, the overseeing RSU initiates the “Video Clip Preloading” module. This module’s role is to orchestrate other autonomous vehicles, ensuring the preloading of the requisite video clips. This guarantees their availability prior to the commencement of the streaming service playback. The autonomous vehicle utilizing the streaming service is also informed about the status of these preloaded video clips.
Step 11: If the preload arrangements made in the previous step do not adequately cater to the playback demands of all video clips, proceed to Step 13 for further execution.
Step 12: This module operates in a background execution mode.
  If there are any alterations to the autonomous vehicle passenger’s route or substantial deviations in the estimated time of arrival at different route segments, the process should revert back to Step 9 for further execution.
Step 13: If the streaming service resolution has been lowered to the system-defined lower limit, this module notifies the autonomous vehicle passenger and returns to Step 12 to continue execution.
Step 14: Decrease the resolution of the streaming service according to the following equation:
  (30) $${sbr}_{s^{\sigma ,\alpha }}^{\sigma ,\alpha }=\left\{\begin{array}{cc} {br}_{q^{\sigma ,\alpha }-1}^{\sigma ,\alpha } & if\;{1\leq q}^{\sigma ,\alpha },\;1\leq s^{\sigma ,\alpha }\leq S^{\sigma ,\alpha }\;\\ {\underline{br}}^{\alpha } & else \end{array},\right.$$
  where ${\underline{br}}^{\alpha }$ represents the system-defined lower limit of video resolution. This module notifies the autonomous vehicle passenger and returns to Step 9 to continue execution.

The computational overhead in this module is primarily driven by the real-time bandwidth allocation or preload planning of video segments for the streaming service application, based on the characteristics and specification demands of the streaming service. Since Step 6 and Step 10 in this module respectively invoke the “Real-Time Streaming Bandwidth Allocation” and “Video Clip Preloading” modules to perform the aforementioned tasks, the computational overhead is determined by the maximum execution time of these two modules. Furthermore, this process may be iterated for different resolution levels, as indicated in Step 14. Consequently, the complexity of this module can be estimated as the maximum execution time required by the two modules mentioned above, multiplied by the number of resolution levels.

### 2.4. Real-Time Streaming Bandwidth Allocation at the RSU

When the RSU receives bandwidth requests for real-time streaming applications from autonomous vehicle passengers with scheduled arrivals at its managed segment, it initiates a preliminary evaluation. This assessment is conducted to verify whether the bandwidth provided by the base stations along the traversed segment is sufficient to meet the requirements of the streaming service. Should the available bandwidth upon the autonomous vehicle’s arrival at the managed RSU segment prove inadequate for the streaming service requirements, this module engages in a reallocation process. It reassigns the previously designated bandwidth, which was initially allocated for concurrent on-demand streaming or 360-degree streaming preloads occurring during the same timeframe. This reallocation is aimed at accommodating the exigencies of the real-time streaming requirement.

If, despite the mentioned bandwidth reassignment, the requirement for real-time streaming bandwidth remains unmet, the RSU employs an alternative approach. It establishes connections between the autonomous vehicle and other vehicles that have arrived at the same segment and are within the range of V2V communication. Through the establishment of a cooperative vehicle fleet, the final vehicle within the fleet acquires sufficient bandwidth from the base stations capable of meeting the streaming service requirements. Subsequently, the harvested bandwidth is transmitted from the last vehicle in the fleet to the requested vehicle via V2V communication. This collaborative strategy guarantees that the requested autonomous vehicle receives the essential bandwidth to effectively support the streaming service for its passenger, thereby enhancing the overall streaming experience.

The steps executed by this module are as follows.

Step 1: This module gives precedence to distributing the remaining bandwidth of base stations within the coverage area of the autonomous vehicle’s route to the specific real-time streaming service. If the service is a videotelephony, the module also allocates any unused upload bandwidth of the base stations to facilitate that videotelephony. If the available base station bandwidth is sufficient to fulfill the real-time streaming service’s bandwidth requirement, the module completes its execution. If not, it moves to the next step.
Step 2: This module reallocates the originally designated bandwidth, which is initially intended for other on-demand streaming or 360-degree streaming preloads occurring within the same time frame, to address the bandwidth requirements of the essential real-time streaming.
Step 3: If the revised allocation effectively satisfies the bandwidth prerequisites of the real-time streaming service, this module advances to Step 6. However, if the adjusted allocation falls short of fulfilling the requirements, this module proceeds to the subsequent step in the sequence.
Step 4: When the bandwidth needed for the real-time streaming service remains insufficient, the RSU communicates with any autonomous vehicle traversing the same road segment within the designated time frame. Through established connections between the vehicle utilizing the streaming service application and other autonomous vehicles, this module utilizes the equations listed below to identify base stations capable of providing the required bandwidth for the real-time streaming service. By harnessing the interconnections within this fleet of vehicles, the essential bandwidth is relayed from base stations possessing sufficient bandwidth capabilities to the passenger’s autonomous vehicle using V2V communication. This collaborative strategy facilitates the delivery of the required bandwidth for the real-time streaming service.
  (31) $${sbr}_{s^{\sigma ,\alpha }}^{\sigma ,\alpha }=\left\{\begin{array}{cc} {br}_{q^{\sigma ,\alpha }-1}^{\sigma ,\alpha } & if\;{1\leq q}^{\sigma ,\alpha },\;1\leq s^{\sigma ,\alpha }\leq S^{\sigma ,\alpha },\;\\ {\underline{br}}^{\alpha } & else \end{array}\right.$$
  subject to:
  (32) $$R_{p_{k}^{\sigma }}^{\sigma ,\alpha }=\left[\left(r_{p_{k}^{\sigma },1}^{\sigma ,\alpha },r_{p_{k}^{\sigma },2}^{\sigma ,\alpha }\right),\cdots ,\left(r_{p_{k}^{\sigma },f-1}^{\sigma ,\alpha },r_{p_{k}^{\sigma },f}^{\sigma ,\alpha }\right),\cdots ,\left(r_{{p_{k}^{\sigma },}^{\sigma ,\alpha }-1}^{\sigma ,\alpha },r_{{p_{k}^{\sigma },}^{\sigma ,\alpha }}^{\sigma ,\alpha }\right)\right],\;\;\;1\leq f<P_{k}^{\sigma ,\alpha },\;1\leq k<h^{\sigma }$$
  (33) $$r_{{p_{k}^{\sigma },}^{\sigma ,\alpha }}^{\sigma ,\alpha }=\sigma ,\;\;1\leq k<h^{\sigma }$$
  (34) $${at}_{p_{1}^{\sigma }}\leq t_{1}^{\sigma ,\alpha }\leq t_{l}^{\sigma ,\alpha }\leq t_{e^{\sigma ,\alpha }}^{\sigma ,\alpha }\leq {at}_{p_{h^{\sigma }}^{\sigma }},\;\;\;\;1\leq l\leq e^{\sigma ,\alpha }$$
  (35) $${at}_{p_{k}^{\sigma }}\leq t_{l}^{\sigma ,\alpha }<{at}_{p_{k+1}^{\sigma }},\;\;1\leq l\leq e^{\sigma ,\alpha },\;\;1\leq k<h^{\sigma }$$
  (36) $$\sum_{\vartheta^{\prime }}{ub}_{t_{l}^{\sigma ,\alpha }}^{\vartheta^{\prime },r_{p_{k}^{\sigma },1}^{\sigma ,\alpha },\alpha }\geq \underline{UR}\left({uvr}_{t_{l}^{\sigma ,\alpha }}^{\sigma ,\alpha }\right)>\sum_{\vartheta }{ub}_{t_{l}^{\sigma ,\alpha }}^{\vartheta ,\sigma ,\alpha },\;\;\;\;1\leq k<h^{\sigma },if\;\gamma^{\sigma ,\alpha }=1\;\;$$
  (37) $$\sum_{\vartheta^{\prime }}{db}_{t_{l}^{\sigma ,\alpha }}^{\vartheta^{\prime },r_{p_{k}^{\sigma },1}^{\sigma ,\alpha }}\geq \underline{DR}\left({dvr}_{t_{l}^{\sigma ,\alpha }}^{\sigma ,\alpha }\right)>\sum_{\vartheta }{db}_{t_{l}^{\sigma ,\alpha }}^{\vartheta ,\sigma ,\alpha },\;1\leq k<h^{\sigma }$$
  (38) $${ut}_{t_{l}^{\sigma ,\alpha }}^{r_{p_{k}^{\sigma },f}^{\sigma ,\alpha },r_{p_{k}^{\sigma },f+1}^{\sigma ,\alpha }}\geq \underline{UR}\left({uvr}_{t_{l}^{\sigma ,\alpha }}^{\sigma ,\alpha }\right)>\sum_{\vartheta }{ub}_{t_{l}^{\sigma ,\alpha }}^{\vartheta ,\sigma },\;\;\;1\leq f<P_{k}^{\sigma ,\alpha },\;1\leq k<h^{\sigma },\;\;if\;\gamma^{\sigma ,\alpha }=1\;$$
  (39) $${dt}_{t_{l}^{\sigma ,\alpha }}^{r_{p_{k}^{\sigma },f}^{\sigma ,\alpha },r_{p_{k}^{\sigma },f+1}^{\sigma ,\alpha }}\geq \underline{DR}\left({dvr}_{t_{l}^{\sigma ,\alpha }}^{\sigma ,\alpha }\right)>\sum_{\vartheta }{db}_{t_{l}^{\sigma ,\alpha }}^{\vartheta ,\sigma },\;\;\;1\leq f<P_{k}^{\sigma ,\alpha },\;1\leq k<h^{\sigma }$$
  where the binary flag $\gamma^{\sigma ,\alpha }=1$ signifies that the streaming service application α is a videotelephony. $p_{k}^{\sigma }$ represents the road segment with insufficient bandwidth. $R_{p_{k}^{\sigma }}^{\sigma ,\alpha }$ represents the fleet initiated from the requested autonomous vehicle on road segment $p_{k}^{\sigma }$, and $P_{k}^{\sigma ,\alpha }$ denotes the number of vehicles within the established fleet. $x_{r_{p_{k}^{\sigma },f}^{\sigma ,\alpha }}$ and $y_{r_{p_{k}^{\sigma },f}^{\sigma ,\alpha }}$ stand for the coordinates of the *f*-th vehicle within the established fleet. ${ut}_{t_{l}^{\sigma ,\alpha }}^{r_{p_{k}^{\sigma },f+1}^{\sigma ,\alpha },r_{p_{k}^{\sigma },f}^{\sigma ,\alpha }}$ and ${dt}_{t_{l}^{\sigma ,\alpha }}^{r_{p_{k}^{\sigma },f+1}^{\sigma ,\alpha },r_{p_{k}^{\sigma },f}^{\sigma ,\alpha }}$, respectively, denote the upload and download bandwidth between autonomous vehicles $r_{p_{k}^{\sigma },f}^{\sigma ,\alpha }$ and $r_{p_{k}^{\sigma },f+1}^{\sigma ,\alpha }$ at time $t_{l}^{\sigma ,\alpha }$.
Step 5: The results of the bandwidth relay are sent back to the initiating autonomous vehicle and the execution of this module is finalized.
Step 6: If the allocation of bandwidth from the base stations to real-time streaming within the same time slot results in video clips for other on-demand streaming or 360-degree streaming being unable to be preloaded promptly, this module triggers the “Video Clip Preloading” module. This activation aims to aid in meeting the preloading prerequisites for the pertinent supported video clips.

The main computational burden in this module arises during Step 4, which involves organizing a fleet of vehicles to transfer essential bandwidth to the passenger’s vehicle through V2V communication from base stations equipped with sufficient bandwidth capabilities. Since bandwidth is relayed from base stations located in the less congested outlying areas of the metropolitan region to autonomous vehicles navigating congested road segments in the central metropolitan region, the maximum distance for bandwidth relay along a path is determined by the longest distance a vehicle travels between the two farthest points in the outlying areas of the metropolitan region.

### 2.5. Video Clip Preloading at the RSU

When confronted with limited bandwidth for on-demand streaming or 360-degree streaming, the RSU initially assesses the road segments along the route traversed by the streaming-enabled autonomous vehicle before the occurrence of the bandwidth deficiency. It explores the presence of base stations within the autonomous vehicle’s route coverage area capable of allocating the necessary bandwidth for preloading the required video clips. 

In situations where the base stations along the route cannot provide the necessary bandwidth, the system turns to other autonomous vehicles that are passing through the same road segments within the streaming service’s operational timeframe. The objective is to identify the base stations capable of providing the necessary capacity for preloading the essential video clips. 

Once a suitable base station is pinpointed, the RSU selects an autonomous vehicle capable of accessing the coverage range provided by that specific base station. This chosen vehicle is then tasked with preloading the required video clips from the identified base station. The preloaded video clip is subsequently stored in the onboard storage of the autonomous vehicle responsible for the preloading. As a result, when the autonomous vehicle that performed the preloading and the autonomous vehicle requesting the video clip come into proximity, the preloaded video clip is sent to the inquiring vehicle via V2V communication.

The following steps are executed by this module:

Step 1: Recognizing that the base stations along the route segment of the autonomous vehicle are insufficient to meet the playback requirements for video clip requested by the vehicle’s passenger, the RSU initiates an investigation into road segments the autonomous vehicle has previously passed through. The aim is to identify base stations within the coverage area that possess sufficient bandwidth to facilitate the preloading of essential video clips for the passenger of the autonomous vehicle.
  (40) $$\sum_{\mu }\left(∆\cdot \sum_{\vartheta^{\prime }}{db}_{{\tau \prime }_{s^{\sigma ,\alpha }}^{\sigma ,\alpha }+\mu \cdot ∆}^{\vartheta^{\prime },\sigma ,\alpha }\right){\geq SS}_{s^{\sigma ,\alpha }}^{\sigma ,\alpha }\left({sbr}_{s^{\sigma ,\alpha }}^{\sigma ,\alpha },{sel}_{s^{\sigma ,\alpha }}^{\sigma ,\alpha }\right),\;\;1\leq s^{\sigma ,\alpha }\leq S^{\sigma ,\alpha }$$
  (41) $$s{el}_{s^{\sigma ,\alpha }}^{\sigma ,\alpha }={el}_{s^{\sigma ,\alpha }}^{\sigma ,\alpha },\;1\leq s^{\sigma ,\alpha }\leq S^{\sigma ,\alpha }$$
  (42) $${pt}_{1}^{\sigma ,\alpha }\leq {\tau \prime }_{s^{\sigma ,\alpha }}^{\sigma ,\alpha }<\tau_{s^{\sigma ,\alpha }}^{\sigma ,\alpha }+\mu \cdot ∆<{pt}_{s^{\sigma ,\alpha }}^{\sigma ,\alpha },\;1\leq s^{\sigma ,\alpha }\leq S^{\sigma ,\alpha }$$
  (43) $${buf}_{{\tau \prime }_{s^{\sigma ,\alpha }}^{\sigma ,\alpha }}^{\sigma }+{SS}_{s^{\sigma ,\alpha }}^{\sigma ,\alpha }\left({br}_{s^{\sigma ,\alpha }}^{\sigma ,\alpha },{sel}_{s^{\sigma ,\alpha }}^{\sigma ,\alpha }\right)\leq {\bar{buf}}^{\sigma },\;\;{pt}_{1}^{\sigma ,\alpha }\leq {\tau \prime }_{s^{\sigma ,\alpha }}^{\sigma ,\alpha }<{pt}_{s}^{\sigma ,\alpha },\;\;\;\;1<s^{\sigma ,\alpha }\leq S^{\sigma ,\alpha },$$
  where ${\tau \prime }_{s^{\sigma ,\alpha }}^{\sigma ,\alpha }$ represents the time at which autonomous vehicle σ traveling through the base station $\vartheta^{\prime }$, which has the capability to supply the necessary bandwidth for preloading the video clip.
Step 2: If any base stations capable of furnishing adequate bandwidth for preloading the required video clip before playback are identified along the vehicle’s route, the relevant RSU overseeing that specific route segment is informed, and the execution of this module comes to an end. If no such base stations are located, then we advance to the subsequent step.
Step 3: On the route of autonomous vehicle σ, we evaluate whether other autonomous vehicles traveling through the same segments and arriving simultaneously possess the capability to conduct preloading of the video clip from base stations positioned along the vehicle’s path. If a vehicle capable of preloading the clip is identified, the clip is preloaded and subsequently transmitted to σ when the two autonomous vehicles meet:
  (44) $$\sum_{\mu }\left(∆\cdot \sum_{\vartheta^{\prime }}{db}_{{\tau \prime }_{s^{\sigma ,\alpha }}^{\sigma ,\alpha }+\mu \cdot ∆}^{\vartheta^{\prime },\sigma ,\alpha }\right){\geq SS}_{s^{\sigma ,\alpha }}^{\sigma ,\alpha }\left({sbr}_{s^{\sigma ,\alpha }}^{\sigma ,\alpha },{sel}_{s^{\sigma ,\alpha }}^{\sigma ,\alpha }\right),\;\;1\leq s^{\sigma ,\alpha }\leq S^{\sigma ,\alpha }$$
  (45) $${pt}_{1}^{\sigma ,\alpha }\leq {\tau \prime }_{s^{\sigma ,\alpha }}^{\sigma ,\alpha }<\tau_{s^{\sigma ,\alpha }}^{\sigma ,\alpha }+\mu \cdot ∆<{pt}_{s^{\sigma ,\alpha }}^{\sigma ,\alpha },\;1\leq s^{\sigma ,\alpha }\leq S^{\sigma ,\alpha }$$
  (46) $${buf}_{{\tau \prime }_{s^{\sigma ,\alpha }}^{\sigma ,\alpha }}^{\rho }+{SS}_{s^{\sigma ,\alpha }}^{\sigma ,\alpha }\left({sbr}_{s^{\sigma ,\alpha }}^{\sigma ,\alpha },{sel}_{s^{\sigma ,\alpha }}^{\sigma ,\alpha }\right)\leq {\bar{buf}}^{\rho },\;\;{pt}_{1}^{\sigma ,\alpha }\leq {\tau \prime }_{s^{\sigma ,\alpha }}^{\sigma ,\alpha }<{pt}_{s}^{\sigma ,\alpha },\;\;\;\;1<s^{\sigma ,\alpha }\leq S^{\sigma ,\alpha }$$
  (47) $${dt}_{{\tau \prime }_{s^{\sigma ,\alpha }}^{\sigma ,\alpha }}^{\rho ,\sigma }\geq \frac{{SS}_{s^{\sigma ,\alpha }}^{\sigma ,\alpha }\left({sbr}_{s^{\sigma ,\alpha }}^{\sigma ,\alpha },{sel}_{s^{\sigma ,\alpha }}^{\sigma ,\alpha }\right)}{\mu \cdot ∆},\;{\;pt}_{1}^{\sigma ,\alpha }\leq {\tau \prime }_{s^{\sigma ,\alpha }}^{\sigma ,\alpha }<{pt}_{s}^{\sigma ,\alpha },\;\;\;\;1<s^{\sigma ,\alpha }\leq S^{\sigma ,\alpha },$$
  where *ρ* represents the supporting autonomous vehicle for clip preloading, and ${\tau \prime }_{s^{\sigma ,\alpha }}^{\sigma ,\alpha }$ stands for the time when *ρ* travels along the road segment covered by base station $\vartheta^{\prime }$, which has the ability to supply the necessary bandwidth for *ρ* to preload the video clip.
Step 4: We send the outcomes of the preload arrangement from the preceding step back to the autonomous vehicle that initiated the bandwidth request and finalize the execution of this module.

The primary computational overhead in this module arises in Step 1 and Step 3, where the RSU endeavors to locate a suitable base station for the preloading of video clips needed for on-demand streaming or 360-degree streaming. Its complexity is calculated by multiplying the number of vehicles simultaneously reaching the same road segment by the number of road segments they may traverse along the longest possible route.

## 3. Simulation Results and Discussion

This study conducted a series of simulations to evaluate the effectiveness of the proposed algorithm. The simulations were performed on a personal computer equipped with an Intel Core i9 5.2 GHz CPU and 128 GB of RAM. The simulation code was developed using Python. In our simulation, traffic density data from a website providing information about traffic in New York City [36] was used to calculate the average speed of autonomous vehicles in each segment, following the equation derived in [37]. Random generation was introduced for both the source and destination of each autonomous vehicle. The total number of vehicles on the roadways throughout the day was synchronized with the traffic density data from [36]. Given the absence of a dedicated autonomous vehicle database, the vehicles mentioned in [36] were considered as autonomous vehicles in our simulation.

The foundation of this study relies on categorizing the multimedia streaming applications used by passengers in autonomous vehicles into four types: videotelephony, live streaming, 360-degree streaming, and on-demand streaming. As the vehicles were in motion, the streaming applications requested by autonomous vehicle users were initiated randomly. The counts and ratios for various applications were determined through an analysis of the four types of streaming applications documented in [38,39,40,41,42,43,44]. Each type of streaming application has distinct bandwidth requirements. Table 1 displays the required download and upload bandwidth for videotelephony [45], while Table 2 outlines the required bandwidth for live streaming [46]. Table 3 elaborates on the required bandwidth for on-demand streaming [47], and Table 4 specifies the bandwidth requirements for 360-degree streaming [48].

The hyperparameters utilized in the proposed algorithm are listed below. For the BILSTM model, following the methodology outlined in [49], we configured the number of neurons in each hidden layer to be 24, set the training epochs to 50, established the input length as 12, determined the batch size as 32, set the learning rate to 0.001, and applied the tanh(∙) activation function for the fully connected layer. Consistent with the approach detailed in [50], we set the time slot interval to 10 s. Each segment’s duration was set to 1000 milliseconds, in line with the recommendation in [51], and each autonomous vehicle was assumed to possess storage space significantly larger than the preloaded segment size. Furthermore, the passenger count within each autonomous vehicle was subject to variation, with possible values ranging from 1 to 4. 

Within the simulated region, an array of base station categories was deployed, including terahertz [52], NLOS-FSOC [53], and sub-6 GHz [54]. These base stations were strategically positioned to cater to the bandwidth requisites of the streaming applications. Table 5 displays the bandwidth capacities and transmission ranges provided by each of these types of base stations. Within the realm of V2V communication, this study conducts simulations involving the utilization of VLC through car lights. [55]. Table 6 showcases the diverse VLC bandwidths at varying distances, discerning between nighttime and daytime scenarios.

**Figure 4:**
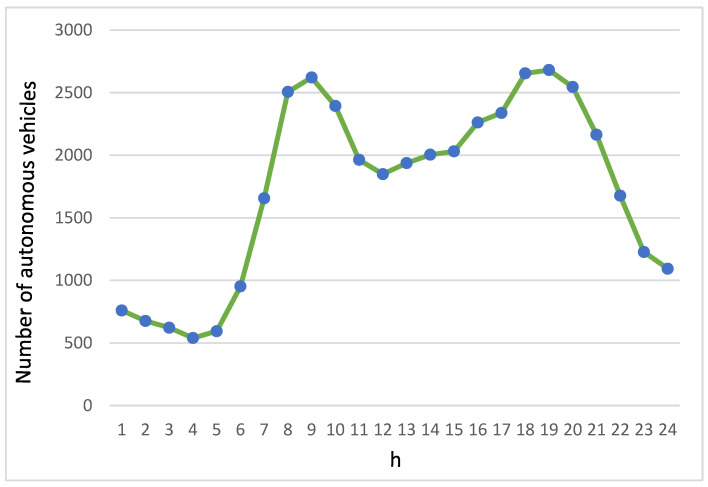
Volume of autonomous vehicles throughout a day.

Figure 4 illustrates the fluctuations in the quantity of autonomous vehicles on the roadway across a day. Notably, during both the morning and evening rush hours, traffic volume significantly increases. The tally of autonomous vehicles commences its rise at 05:00 in the morning, reaching its climax around 09:00 during the morning peak. Following this peak phase, the flow of vehicles stabilizes at roughly 2000 vehicles. Subsequent to 16:00, there is a gradual increase in the number of autonomous vehicles, reaching its pinnacle around 19:00 in the evening, succeeded by a reduction in traffic volume. The lowest number of autonomous vehicles was observed during the very early hours of the morning.

**Figure 5:**
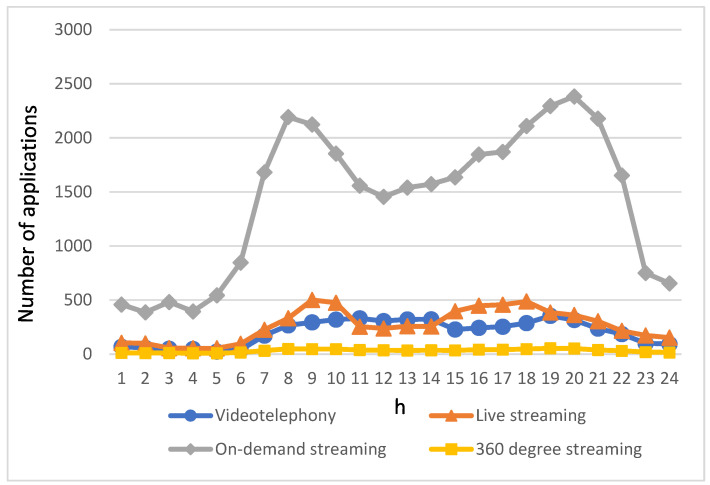
Counts of applications initiated by passengers in autonomous vehicles throughout a day.

Figure 5 illustrates the usage of applications by passengers in autonomous vehicles throughout the day, categorized into four types: videotelephony, live streaming, on-demand streaming, and 360-degree streaming. It is noteworthy that the application usage patterns closely mirror the fluctuations in the number of autonomous vehicles, as shown in Figure 4. In the early morning hours when traffic is light, there is relatively low usage of streaming service applications. However, as morning and evening rush hours approach, there is a significant surge in the usage of various streaming applications. This is particularly pronounced as traffic congestion leads to extended commute times, prompting passengers in autonomous vehicles to have a greater demand for streaming service applications.

Remarkably, on-demand streaming remains the preferred choice for passengers due to its flexibility in viewing schedules, content replayability, and wide range of content options. This preference holds true during both peak and off-peak hours. In contrast, the availability of 360-degree streaming services is relatively limited. This scarcity is not only due to the limited prevalence of devices capable of viewing 360-degree streaming but also because the quantity of 360-degree streaming content available on streaming platforms is far from matching the abundance of on-demand streaming content.

**Figure 6:**
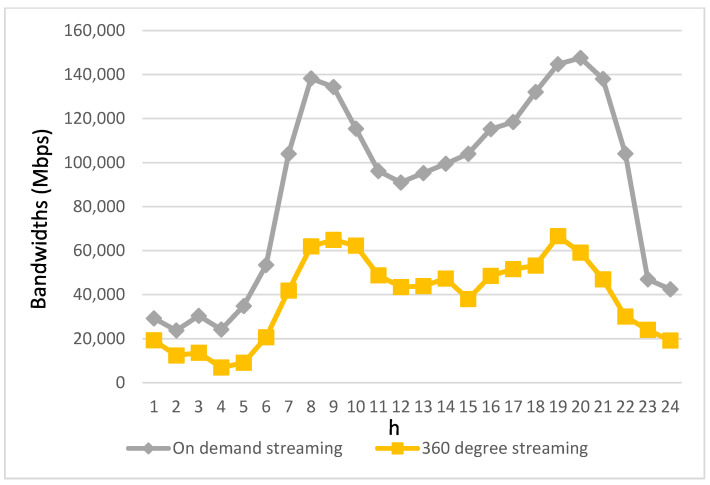
Bandwidth requirements for on-demand streaming and 360-degree streaming within a day.

**Figure 7:**
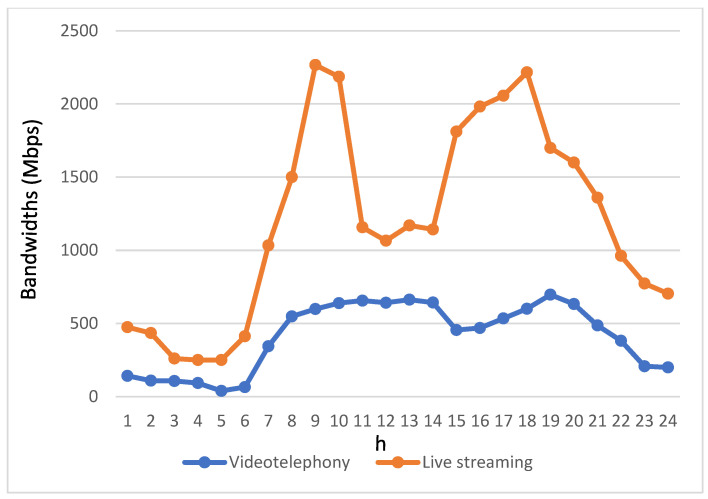
Bandwidth requirements for videotelephony and live streaming within a day.

Figure 6 and Figure 7 provide insights into the bandwidth requirements of various service applications used by passengers in autonomous vehicles throughout the day. The bandwidth demands of these four streaming applications are directly proportional to their usage levels. Notably, due to the relatively modest usage of videotelephony and live streaming, their bandwidth requirements are notably lower. Therefore, the depiction of bandwidth requirements for videotelephony and live streaming is presented separately from the other two streaming applications to avoid confusion when comparing variations in these two applications with the others in a single figure.

As shown in Figure 6, it becomes evident that on-demand streaming exhibits significantly greater bandwidth requirements compared to other streaming applications. This can be attributed to its larger user base and the substantial bandwidth demands of high-resolution streaming content. It is worth noting that despite having a considerably lower user count compared to other streaming applications, 360-degree streaming aims to deliver an immersive and lifelike visual experience, necessitating substantial bandwidth resources to transmit lifelike three-dimensional content. Therefore, Figure 6 underscores the high bandwidth requirements of 360-degree streaming.

Regarding the bandwidth requirements of videotelephony and live streaming, Figure 7 reveals that live streaming requires a higher bandwidth allocation compared to videotelephony. This contributes to an elevated overall bandwidth demand for live streaming throughout the day.

**Figure 8:**
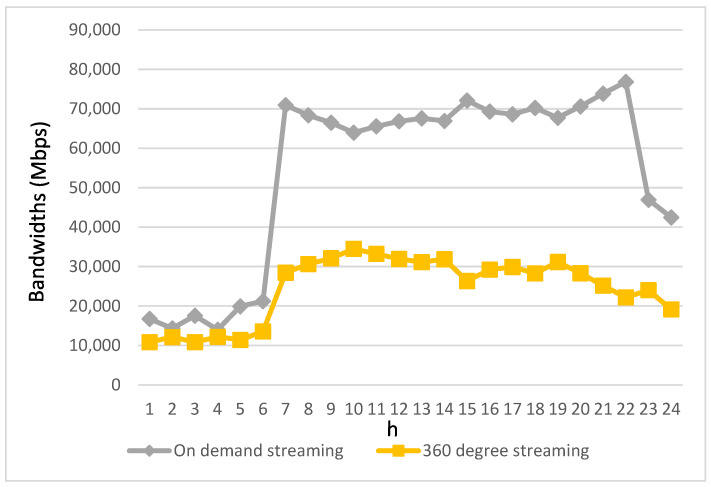
Bandwidth allocation for on-demand streaming and 360-degree streaming prior to the algorithm’s implementation.

**Figure 9:**
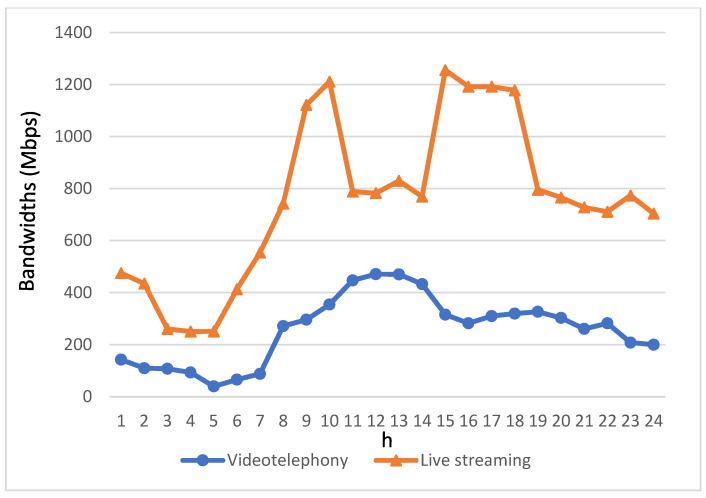
Bandwidth allocation for videotelephony and live streaming applications prior to the algorithm’s implementation.

Figure 8 and Figure 9 illustrate the allocation of bandwidth for each type of streaming application prior to the implementation of the proposed algorithm. In contrast to Figure 6 and Figure 7, which depict the bandwidth requirements of different types of streaming applications, it can be observed that during periods when the bandwidth requirements for various streaming applications are relatively low, the allocated bandwidth closely aligns with their respective needs. However, as time progresses and the bandwidth demands of these applications increase, the allocated bandwidth starts to fall short of meeting the required bandwidth. This disparity becomes more pronounced, especially during peak intervals characterized by a significant increase in bandwidth demands from the streaming applications.

**Figure 10:**
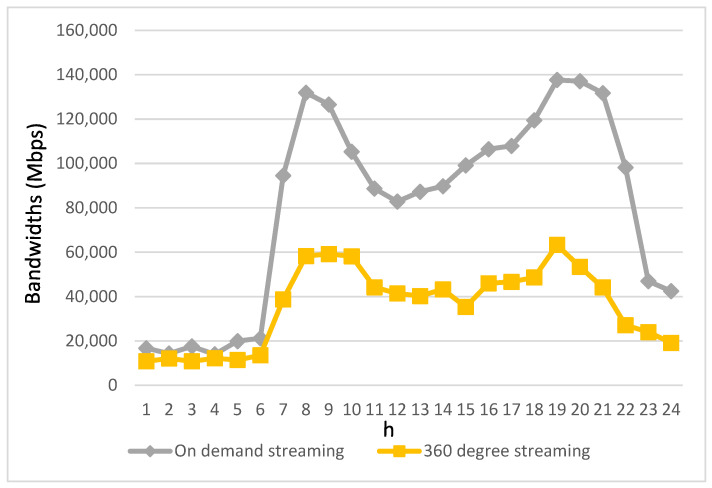
Bandwidth allocated for applications of on-demand streaming and 360-degree streaming after applying the proposed algorithm.

**Figure 11:**
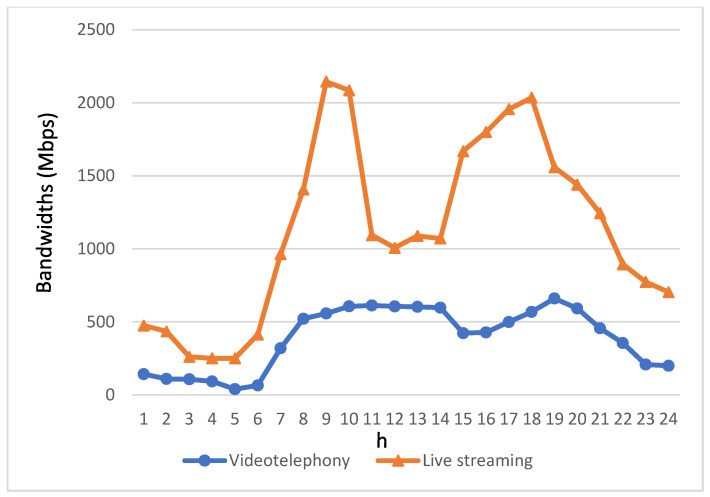
Bandwidth allocated for applications of videotelephony and live streaming after applying the proposed algorithm.

Figure 10 and Figure 11 illustrate the resulting bandwidth allocation for various applications following the implementation of the algorithm proposed in this study. As anticipated, the algorithm ensures that each type of multimedia streaming application receives bandwidth resources tailored to its specific requirements. Notably, during periods of high bandwidth demand, there is a discernible increase in the available bandwidth.

In scenarios where the bandwidth requirements of streaming applications for autonomous vehicle passengers cannot be fully met, the proposed “Streaming Service Support” module comes into play. This module dynamically adjusts the bandwidth requirements and quality of the streaming service to address disparities between the supply and demand for bandwidth, particularly during peak usage times. Additionally, the “Real-time Streaming Bandwidth Allocation” module prioritizes the redistribution of the initially allocated bandwidth, originally designated for non-real-time applications, to support real-time videotelephony and live streaming demands. Alternatively, through V2V communication, the bandwidth supplied by base stations located on other road segments can be relayed to facilitate seamless videotelephony and live streaming for users. Specifically, after implementing the proposed algorithm, the bandwidth for real-time videotelephony and live streaming applications improves by 73% and 60%, respectively, during the high-demand periods from 7:00 a.m. to 10:00 p.m.

Regarding on-demand streaming and 360-degree streaming, the “Video Clip Preloading” module is employed to preload video clips for autonomous vehicle passengers before their playback times. In cases where these vehicles converge on the same road segment as the supported autonomous vehicles facing bandwidth limitations for on-demand and 360-degree streaming, video clips can be preloaded to the conveying vehicles within the coverage of base stations with sufficient bandwidth. The preloaded video clips can then be transmitted to the requesting autonomous vehicle passengers for playback through V2V communication. Consequently, bandwidth availability for on-demand and 360-degree streaming sees improvements, with increases of 58% and 57%, respectively, during the high-demand periods from 7:00 a.m. to 10:00 p.m. This improvement underscores the effectiveness of this work in prioritizing real-time applications and optimizing non-real-time streaming applications through the proposed preloading strategies.

**Figure 12:**
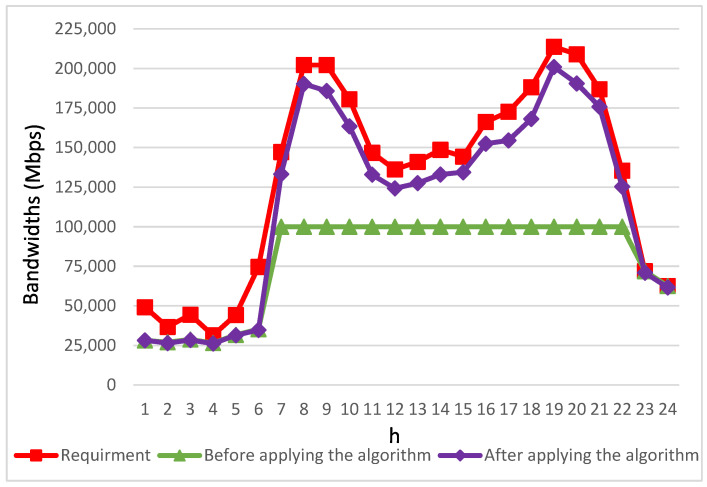
User-required bandwidth and the bandwidth available before and after applying the algorithm.

Figure 12 presents a comparison between the bandwidth requested by various streaming applications and the actual bandwidth allocated before and after the implementation of the proposed algorithm. Between 11:00 p.m. and 6:00 a.m., there is relatively low demand for bandwidth from autonomous vehicle passengers, and almost all applications successfully receive sufficient bandwidth allocation. However, from 7:00 a.m. until 10:00 p.m., prior to the algorithm’s implementation, numerous applications have already reached the maximum bandwidth limit set by the congested road segment’s base stations. Consequently, their bandwidth requirements remain unfulfilled. Moreover, as the number of applications increases, the disparity between the obtained bandwidth and the demanded bandwidth becomes more pronounced.

During the period from 7:00 a.m. to 10:00 p.m., when the base stations on congested road segments had reached their maximum bandwidth capacity, all passengers in autonomous vehicles experienced an average bandwidth improvement of 55%. Furthermore, throughout the two peak demand intervals, in the morning at 8:00 a.m. and in the evening at 7:00 p.m., passengers in autonomous vehicles saw remarkable increases of 90% and 100%, respectively, in the available bandwidth.

This study employs a decentralized computational architecture to address the computational complexities associated with traditional centralized control frameworks. The total computational complexity of the proposed work can be divided into three distinct parts. Firstly, the “Real-Time Vehicle Speed Calculation” module at a RSU continually updates the average vehicle speed as the autonomous vehicle travels through managed segments within specific time intervals. When an autonomous vehicle begins its journey, it utilizes the “Real-Time Driving Speed and Time Calculation” module to determine its driving route.

Following this, when a passenger initiates a streaming service application within the autonomous vehicle, the remaining three modules collaborate to allocate the necessary bandwidth for the streaming service. Specifically, the “Streaming Service Support” module invokes the “Real-Time Streaming Bandwidth Allocation” and “Video Clip Preloading” modules to perform real-time bandwidth allocation or preload planning of video segments for the streaming service application, based on the characteristics and specification demands of the streaming service. Therefore, the maximum computational complexity in this context should be determined as outlined in the last paragraph of the “Streaming Service Support” module.

It is noteworthy that this study introduces an innovative approach to address the potential bandwidth limitations faced by upcoming vehicular networks, especially during periods of high demand. The proposed approach involves a unique strategy: by accurately calculating the estimated arrival times of autonomous vehicles at different segments of their routes, and by leveraging V2V communication, it becomes possible to efficiently distribute bandwidth resources. This redistribution of bandwidth occurs from base stations strategically positioned in less congested road sections to the autonomous vehicles that require additional bandwidth support. To be specific, the proposed algorithm effectively facilitates the delivery of preloaded video clips to autonomous vehicles facing bandwidth inadequacies while navigating congested roads, utilizing V2V communication. This results in a substantial increase in the allocated bandwidth for streaming applications used by autonomous vehicle passengers. Furthermore, it substantially narrows the gap between the acquired and required bandwidth. Last but not least, the proposed algorithm further boosts overall bandwidth utilization by employing bandwidth relay from less congested base stations.

## 4. Conclusions

While the existing literature has laid the foundation for bandwidth allocation algorithms within vehicular networks, there is still an opportunity to explore the untapped potential presented by the emerging next-generation wireless communication technologies within the dynamic context of Autonomous Vehicle Networks. This study is driven by the mission to bridge this gap by seamlessly integrating cutting-edge terahertz and NLOS-FSOC wireless communication technologies with the more conventional sub-6 GHz band base stations. The overarching goal is to ingeniously distribute the necessary bandwidth for streaming applications, catering with precision to the passengers of autonomous vehicles. While the existing literature has paved the way for bandwidth allocation algorithms in vehicular networks, a noticeable void persists in harnessing the untapped potential of emerging 6G wireless communication technologies within the intricate landscape of the Internet of Autonomous Vehicles. This study is dedicated to closing this gap by seamlessly integrating state-of-the-art terahertz and NLOS-FSOC wireless communication technologies with the established sub-6 GHz band base stations. The overarching objective is to strategically allocate the necessary bandwidth for streaming applications, tailored to meet the discerning needs of passengers within autonomous vehicles. To materialize this objective, the study adeptly deploys terahertz and NLOS-FSOC technologies, orchestrating a harmonious coexistence of micro and macro base stations alongside the well-established sub-6 GHz band base stations. An outstanding addition is the strategic incorporation of BILSTM, which serves as the linchpin for the timely and precise allocation of bandwidth. This allocation is intricately aligned with the distinct quality requirements of individual passengers as they embark on their autonomous journeys. Elevating the innovative landscape, the study introduces VLC technology, a pivotal enhancer of both security and data rates in V2V communications. This augmentation significantly raises the bar for the quality of V2V streaming transmissions, consequently elevating the holistic streaming experience. 

The effectiveness of these proposed mechanisms is demonstrated through a comprehensive analysis of the simulation results. The simulation results reveal that the proposed work significantly enhances the available bandwidth for autonomous vehicle passengers during periods when base stations on congested road segments have reached their maximum bandwidth capacity. The results indicate an average bandwidth improvement of 55%, which is accessible to all passengers in autonomous vehicles. As a result, a wide range of streaming applications—encompassing videotelephony, live streaming, on-demand content, and immersive 360-degree experiences—can seamlessly access timely and essential bandwidth support. This holds true even when base station resources face strain during heightened travel demands. In conclusion, the innovative algorithm presented in this study goes beyond mitigating potential bandwidth conflicts among passengers within autonomous vehicles for base station resources. It ensures uninterrupted access to streaming applications, especially during peak demand periods. This comprehensive approach systematically maximizes overall service satisfaction by skillfully addressing the diverse quality needs of the majority of passengers. In doing so, it firmly positions itself at the forefront of bandwidth management within the evolving realm of the Internet of autonomous vehicles. 

Limited by the dataset availability for autonomous vehicles, we have used currently available traffic data of vehicles in our simulations. However, in the future, when real-world datasets of autonomous vehicles become accessible, we intend to employ up-to-date autonomous vehicle datasets for our simulations in future research. Additionally, we plan to broaden the scope of our research to encompass both human-driven and autonomous vehicles within vehicular networks. We aim to develop strategies to address the challenges associated with predicting vehicle travel times in the complex landscape of hybrid vehicular networks, which include both human-driven and autonomous vehicles.

Furthermore, we plan to explore the potential utilization of additional technologies, such as satellites and drones, as sources of bandwidth in the future. Lastly, we are committed to integrating state-of-the-art machine learning predictive streaming content caching systems into our future research endeavors to enhance the overall comprehensiveness and effectiveness of our systems.

## Figures and Tables

**Table 1:** Bandwidth requirements for videotelephony.

Streaming Format	Download Required Bandwidth	Upload Required Bandwidth
high-quality	600 Kbps	600 Kbps
720 p	1.2 Mbps	1.2 Mbps
1080 p	3.0 Mbps	3.8 Mbps

**Table 2:** Bandwidth requirements for live streaming.

Streaming Format	Download Required Bandwidth
720 p 30 fps	3 Mbps
1080 p 30 fps	4.5 Mbps
1080 p 60 fps	6 Mbps

**Table 3:** Bandwidth requirements for on-demand streaming.

Streaming Format	Download Required Bandwidth
720 p 30 fps	5 Mbps
1080 p 30 fps	8 Mbps
1440 p 30 fps	16 Mbps
2160 p 60 fps	68 Mbps

**Table 4:** Bandwidth requirements for 360-degree streaming.

Streaming Format	Download Required Bandwidth
4 K 30 fps	25 Mbps
8 K 30 fps	100 Mbps
12 K 60 fps	400 Mbps
24 K 120 fps	2.35 Gbps

**Table 5:** Bandwidth capacities and transmission ranges offered by the three categories of base stations.

Category of Base Station	Transmission Distance	Bandwidth
Terahertz	39 m	24~54 Gbps
NLOS-FSOC	200 m	1~100 Gbps
Sub-6GHz	622 m	0.5~1 Gbps

**Table 6:** Maximal distance and bandwidth for VLC.

Distance	Max. Bandwidth during Daylight	Max. Bandwidth during the Night
10 m	2790 Mbps	2810 Mbps
100 m	336 Mbps	362 Mbps

## Data Availability

Not applicable.
